# First person – Stephanie Fernandes

**DOI:** 10.1242/dmm.043851

**Published:** 2020-01-10

## Abstract

First Person is a series of interviews with the first authors of a selection of papers published in Disease Models & Mechanisms, helping early-career researchers promote themselves alongside their papers. Stephanie Fernandes is first author on ‘Altered *in vitro* muscle differentiation in X-linked myopathy with excessive autophagy’, published in DMM. Stephanie conducted the research described in this article while a master's degree student in Mariz Vainzof's lab at the Human Genome and Stem-Cell Research Center, University of São Paulo, São Paulo, Brazil. She is now a PhD student in the lab of Constantinos Demetriades at Max Planck Institute for Biology of Ageing, Cologne, Germany, investigating how nutritional status can regulate cell growth in health and age-related disease.


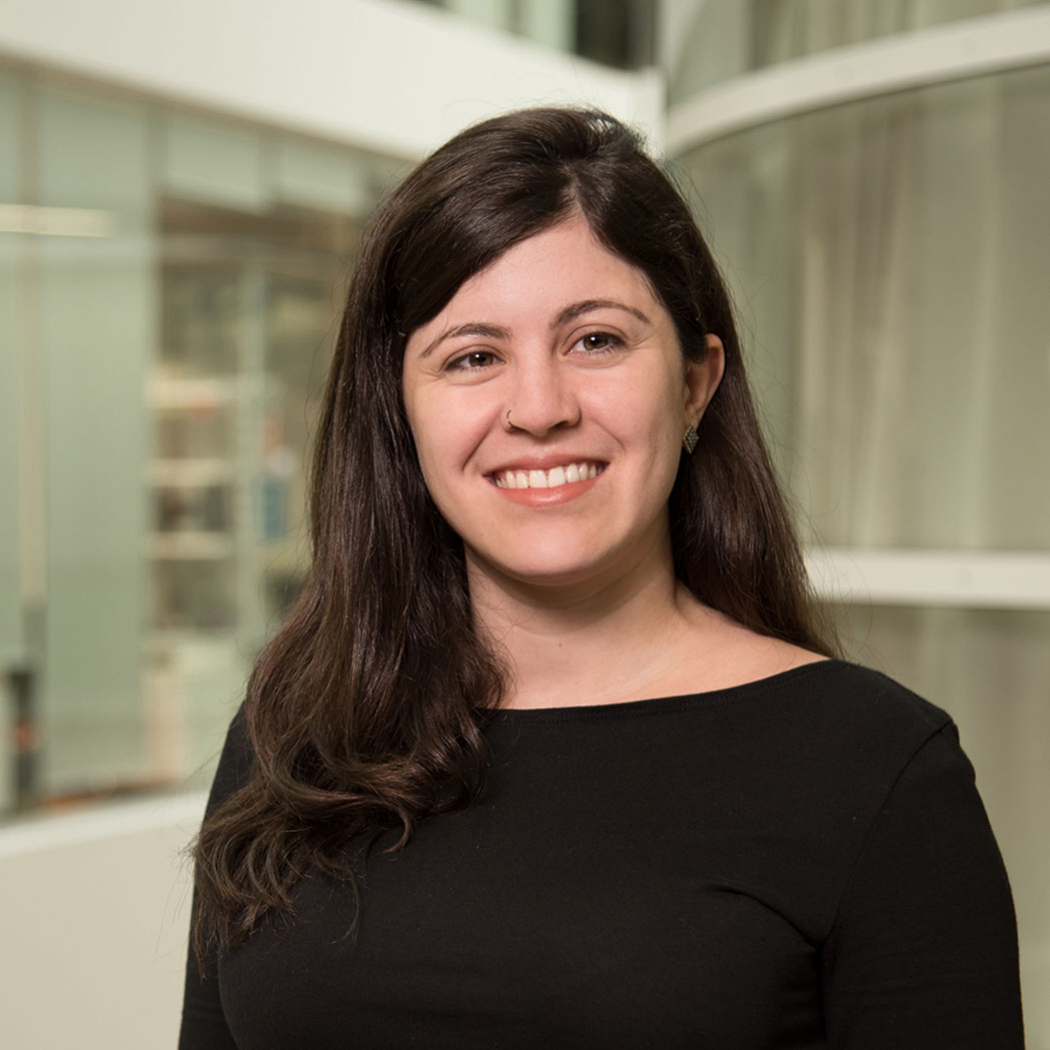


**Stephanie Fernandes**

**How would you explain the main findings of your paper to non-scientific family and friends?**

X-linked myopathy with excessive autophagy (XMEA) is a rare genetic disease in which the muscles of affected boys cannot function properly over time. The disease starts by a mutation that causes one protein to be partially missing from patient cells. However, since this protein is missing from several tissues, it is intriguing how the muscles are the only affected organs. In our work, we established that in the early stages of muscle formation, there is no sign of the disease. However, in later stages, when the disease manifests at the cellular level, surprisingly, we observed an accompanying defect in muscle formation. This could provide a possible explanation for the specific effect on muscles over other tissues.

**What are the potential implications of these results for your field of research?**

We have established the time point at which the features of XMEA begin to manifest. We show that patient-derived progenitor muscle cells do not show features of the disease, such as accumulation of autophagic vacuoles. However, in later stages of myogenesis, we observe not only altered autophagy but also altered muscle differentiation. This points to a possible mechanism by which the primary mutation affects both these processes. In this manner, the partial loss of VMA21, which is a protein that affects lysosomal acidification, could preferentially affect muscles in spite of being ubiquitously expressed, providing a line of evidence regarding the exclusive muscle phenotype. Moreover, the time point at which the changes in muscle formation occur is of utmost importance for any therapeutic intervention, since it provides a time window in which therapies could be successful.

**What are the main advantages and drawbacks of the model system you have used as it relates to the disease you are investigating?**

The model system used in our study is directly derived from a patient biopsy, which makes it a suitable model for studying the phenotypic characteristics of the disease. In addition, the patient-derived cell line allows us to differentiate muscle progenitor cells into multinucleated myotubes. Having such a system allows us to understand how the disease might affect muscle formation. However, a drawback of this system is that *in vitro* muscle formation can be achieved only until a certain stage. We overcame this limitation by using the patient biopsy, which originates from a mature muscle.

“The most exciting and surprising moment was when, after 6 days of differentiating muscle progenitor cells, we could observe myotubes contracting in the culture flask!”

**What has surprised you the most while conducting your research?**

The most exciting and surprising moment was when, after 6 days of differentiating muscle progenitor cells, we could observe myotubes contracting in the culture flask! It showed me how we can recapitulate amazing phenomena even *in vitro*. Moreover, most diseases that affect muscle differentiation and function are related to reduced fusion of muscle progenitor cells. Therefore, it was a surprise to see that, on the contrary, in our system, an increase in fusion of muscle progenitor cells led to a less functional tissue.

**Describe what you think is the most significant challenge impacting your research at this time and how will this be addressed over the next 10 years?**

We believe that *in vitro* models are a valuable tool to understand molecular mechanisms that are linked to genetic diseases. More importantly, having models that have originated from patient samples are an exciting and informative alternative to established cell lines. Nonetheless, there is a gap between the maximum stage of muscle development that can be achieved in an *in vitro* system compared to an *in vivo* mature muscle. In the next 10 years, it will be important to bridge this gap, to fully uncover the different stages of disease progression and its underlying mechanisms.

**Figure:**
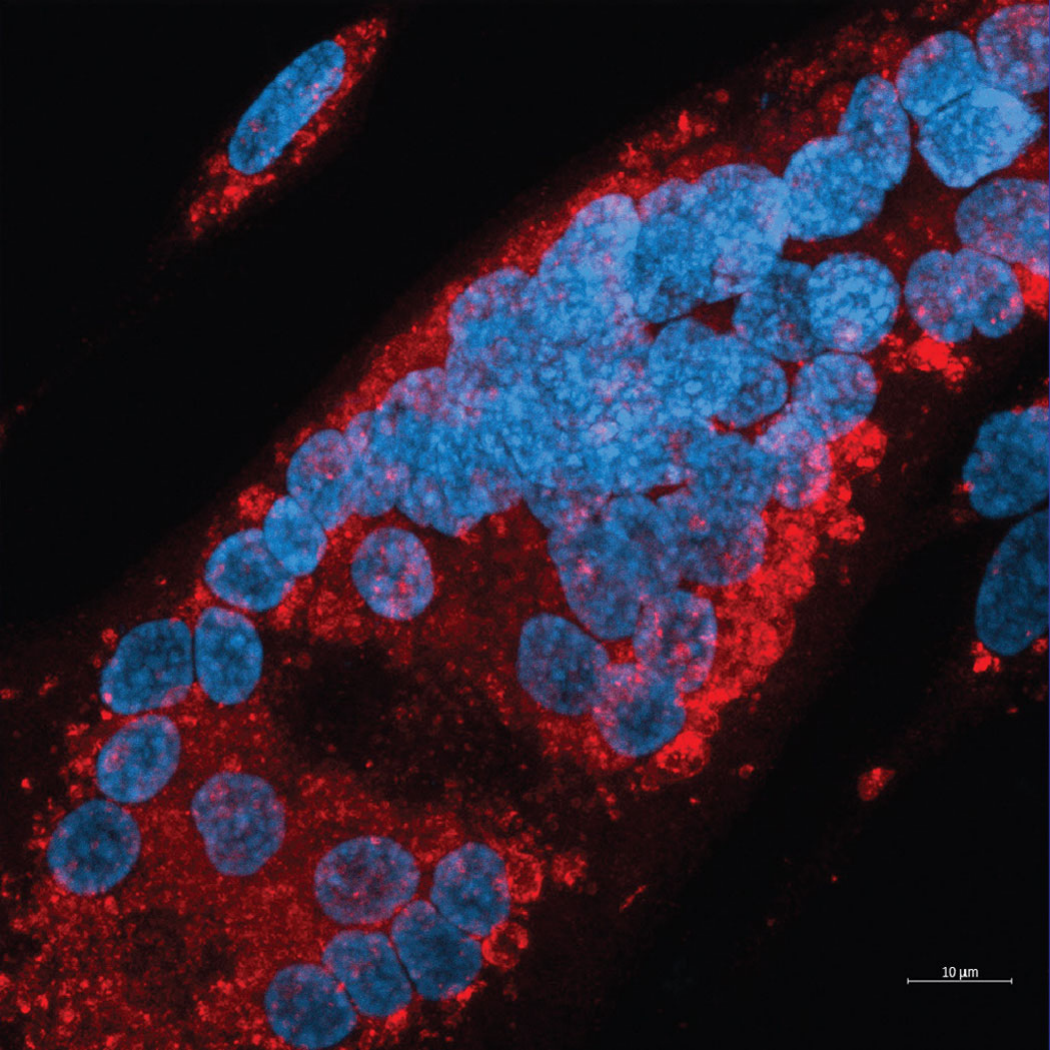
**A large XMEA myotube that contains several nuclei, in blue, and autophagosomes, in red.**

“[…] promoting international experiences in order to broaden one's view of research is of utmost importance for early-career researchers.”

**What changes do you think could improve the professional lives of early-career scientists?**

During my undergraduate studies, I had the opportunity to study in the UK for 1 year as part of an exchange program. During this period, I did an internship in a lab that changed my way of doing and thinking science. Because of that, I believe that promoting international experiences in order to broaden one's view of research is of utmost importance for early-career researchers.

**What's next for you?**

After completing my master's degree studies in Brazil, I moved to Germany, where I am currently pursuing my PhD degree at the Max Planck Institute for Biology of Ageing. Here, once more, I benefit from the international environment and exchange of ideas taking place that make the research experience great. After graduating, I plan to apply for postdoctoral fellowships in the USA, in order to get acquainted with other scientific mindsets.

## References

[DMM043851C1] Fernandes S. A., Almeida C. F., Souza L. S., Lazar M., Onofre-Oliveira P., Yamamoto G. L., Nogueira L., Tasaki L. Y., Cardoso R. R., Pavanello R. C. M. et al. (2020). Altered *in vitro* muscle differentiation in X-linked myopathy with excessive autophagy. *Dis. Model. Mech.* 13, dmm041244 10.1242/dmm.041244 31826868 PMC6994946

